# Characterization of Estrogenic Activity and Site-Specific Accumulation of Bisphenol-A in Epididymal Fat Pad: Interfering Effects on the Endocannabinoid System and Temporal Progression of Germ Cells

**DOI:** 10.3390/ijms22052540

**Published:** 2021-03-03

**Authors:** Teresa Chioccarelli, Marina Migliaccio, Antonio Suglia, Francesco Manfrevola, Veronica Porreca, Nadia Diano, Sonia Errico, Silvia Fasano, Gilda Cobellis

**Affiliations:** 1 Department of Experimental Medicine, Sez. Bottazzi, Università degli Studi della Campania “L. Vanvitelli”, Via Costantinopoli 16, 80138 Napoli, Italy; teresa.chioccarelli@unicampania.it (T.C.); marina.migliaccio83@gmail.com (M.M.); francesco.manfrevola@unicampania.it (F.M.); veronica.porreca@unicampania.it (V.P.); nadia.diano@unicampania.it (N.D.); sonia.errico@unicampania.it (S.E.); silvia.fasano@unicampania.it (S.F.); 2 EHESP, Inserm, Irset (Institut de Recherche en Santé, Environnement et Travail)—UMR_S 1085, Université de Rennes, 35000 Rennes, France; antonio.suglia@univ-rennes1.fr

**Keywords:** bisphenol-A, testis, epididymal fat, germ cell progression, spermatogenesis, endocannabinoids, CB2, 2-AG, blood-testis-barrier

## Abstract

The objective of this work has been to characterize the estrogenic activity of bisphenol-A (BPA) and the adverse effects on the endocannabinoid system (ECS) in modulating germ cell progression. Male offspring exposed to BPA during the foetal-perinatal period at doses below the no-observed-adverse-effect-level were used to investigate the exposure effects in adulthood. Results showed that BPA accumulates specifically in epididymal fat rather than in abdominal fat and targets testicular expression of 3β-hydroxysteroid dehydrogenase and cytochrome P450 aromatase, thus promoting sustained increase of estrogens and a decrease of testosterone. The exposure to BPA affects the expression levels of some ECS components, namely type-1 (CB1) and type-2 cannabinoid (CB2) receptor and monoacylglycerol-lipase (MAGL). Furthermore, it affects the temporal progression of germ cells reported to be responsive to ECS and promotes epithelial germ cell exfoliation. In particular, it increases the germ cell content (i.e., spermatogonia while reducing spermatocytes and spermatids), accelerates progression of spermatocytes and spermatids, promotes epithelial detachment of round and condensed spermatids and interferes with expression of cell–cell junction genes (i.e., zonula occcludens protein-1, vimentin and β-catenin). Altogether, our study provides evidence that early exposure to BPA produces in adulthood sustained and site-specific BPA accumulation in epididymal fat, becoming a risk factor for the reproductive endocrine pathways associated to ECS.

## 1. Introduction

A number of anthropogenic chemical environmental pollutants have been classified as endocrine disruptors (EDs) being they able to interfere with the endocrine system (i.e., hormonal biosynthesis, metabolism and activity) [1,2]. Bisphenol-A (BPA) is considered an endocrine disruptor (ED), preferentially with estrogenic activity, due to its ability to mimic the action of endogenous estrogenic hormones. BPA is able to interact with the estrogen receptor (ER) alpha (αER; with agonistic and antagonistic effects) and beta (βER; with agonistic effects) [3], the G-protein-coupled estrogen receptor GPR30, the estrogen-related receptor gamma ERRγ [4,5,6,7,8] as well as to target androgen receptor signalling [9]. Hence, BPA shows estrogenic and anti-androgenic activities.

BPA is widely spread in the environment, being a highly stable pervasive industrial chemical useful for the production of plastics and epoxy resins commonly used in the manufacture of consumables, food packaging, dental sealants, medical devices and other medical equipment [10]. Due to its extensive use, BPA has been found to contaminate the aquatic environment and foodstuffs, thus it is widely detected in human tissues (adipose and placental) and biological fluids (plasma, blood, urine, breast milk, seminal plasma, saliva, follicular/amniotic fluid), including foetal serum [1].

In animal models, the accumulation of BPA in foetuses and offspring during the foetal/neonatal period has been demonstrated to depend on the maternal exposure and to be mediated by the placenta or breast milk [11,12], as the efficient drug-metabolising systems of the mothers is slightly altered during pregnancy [13,14,15]. This accumulation occurs since the offspring acquire only late after birth the ability to metabolize BPA, quite late after birth [e.g., 21 days post partum (*dpp*) in rat] [16] thus introducing a large measure of uncertainty in any risk assessment that is exclusively based on dose-effect observations, not considering the real accumulation of BPA that also depends on variations in individual susceptibility [17]. Indeed, three hazard doses for BPA exposure were defined: tolerable daily intake (TDI; 50 μg/kg body weight (bw)/day, indicated as safe reference dose for human), no-observed-adverse-effect-level (NOAEL; 5 mg/kg bw/day), and lowest-observed-adverse-effect level (LOAEL; 50 mg/kg bw/day) [18,19].

However, a number of studies describe BPA effects in animals exposed to <50 mg/kg/day, including effects on male reproductive tract referable to early exposure [10,20,21]. Studies highlighting the real early-exposure-dependent BPA accumulation in target tissues associated to adverse reproductive effects are absent.

As an estrogenic chemical, BPA is specifically defined as a selective estrogen-receptor-modulator (SERM) [22]. Consequently, exposure to BPA has been linked to developmental, systemic, reproductive, neurological and immune disorders in both humans and animals [23,24]. Impairment of spermatogenesis and functional sperm parameters, including germ cell proliferation [25], steroidogenesis [26,27,28,29,30,31,32], blood-testicular barrier (BTB) junctions [33,34], acrosome and spermatid differentiation [35], as well as mitochondrial function, chromatin condensation and integrity, number, motility and vitality of spermatozoa (SPZs) [21,36,37,38] are among the main interfering effects of BPA, documented so far in several animal models, including mice and rats, exposed pre/post-birth to low/high doses of BPA [26].

In addition to neuroendocrine control, several testicular modulators and a locally epididymal-fat derived key factor, currently unidentified, affect the progression of spermatogenesis and production of SPZs [39,40]. Interestingly, a cross-talk between endocannabinoids and estrogens has recently been reported in the regulation of hypothalamic-pituitary-gonadal activity [41] and adipose tissue [42]. More interesting is that both modulate either the progression of spermatogensis and the adiposity of epididymal fat (perigonadal epididymal withe tissue, pEWAT) [42,43,44,45], tracing a potential harmful effect of BPA on germ cells which could also result from the impairment of nearby pEWAT. Notably, BPA accumulates in fat tissue [1,12,17,46,47,48,49], it targets gene expression of enzymes metabolizing endocannabinoids [1,50,51,52,53] and steroids [54,55] and its accumulation in fat tissue is related to steroid levels [17].

The endocannabinoids are lipidic mediators. Arachidonoylethanolamine (or anandamide, AEA) and 2-arachidonoylglycerol (2-AG) are the main endocannabinoids characterized in testis and fat mass [56,57,58]. Both AEA and 2-AG are produced “on demand” from membrane phospholipids by NAPE-hydrolyzing phospholipase D (NAPE-PLD) [59] and sn-1-DAG-lipase (DAGL) [60] respectively, while their degradation is controlled by the fatty acid amide hydrolase (FAAH), the AEA/2-AG-degrading enzyme, and monoacylglycerol-lipase (MAGL) that hydrolyses 2-AG [61,62]. Both bind and activate type-1 (CB1) and type-2 (CB2) cannabinoid receptors [63,64] constituting, in combination with endocannabinoid membrane transporters, a cell signalling system known as the endocannabinoid system (ECS).

Endocannabinoids modulate spermatogenesis, including spermatogonia differentation, mitotic-meiotic switch and progression [56,65,66], spermatid differentiation [43,44,45,67,68], steroidogenesis [69,70,71,72,73], specifically synthesis of testosterone (T) and 17-β Estradiol (E2), as well as epididymal sperm motility acquisition and maturation [67,74].

Recent findings reveal that BPA deregulates the ECS in several tissues [42,50,75]. Consistently, we have recently reported that early exposure to BPA doses below the not observed-adverse effect levels (NOEL) value during the foetal/perinatal period, induces anorexigenic phenotype in prepubertal males with effects in adulthood on body weight and pEWAT adiposity [42]. Interestingly, these animals also showed sustained sperm death in *caput* epididymis, with no effects in *cauda* [36]. The molecular characterization of the anorexigenic phenotype in exposed animals revealed that BPA exerted agonistic estrogenic effects and counteracted the endogenous orexigenic activity of the ECS by interfering with the hypothalamic expression of ECS components [42].

Here, using tissues collected from the same batch of animals early exposed to BPA described above [17,36], we have characterized the estrogenic activity of BPA and verified the effects on testicular expression of ECS as a potential cause of impairment of spermatogenesis and sperm death in epididymal *caput*.

## 2. Results

### 2.1. BPA, Testosterone and 17-β-Estradiol Content in Adipose Tissues and Testis

**Figure 1:**
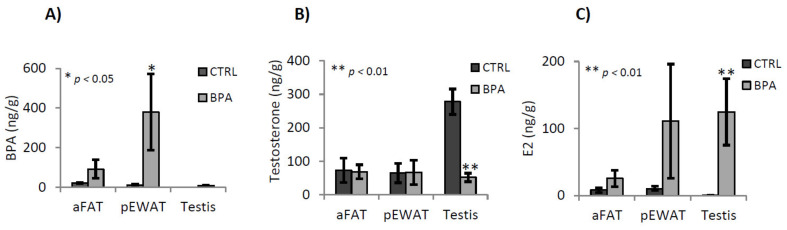
Analysis of BPA (**A**), testosterone (T) (**B**) and 17-beta-estradiol (E2) (**C**) content (as ng/g) in white abdominal (aFAT) and epididymal (pEWAT) adipose tissue and in testis of male mice early-life exposed to vehicle (CTRL, *n* = 3/4) or BPA (BPA, *n* = 4) and sacrificed at 78 *dpp*. All the data were reported as mean value ± S.E.M. (*: *p* < 0.05; **: *p* < 0.01).

Visceral white adipose tissues [differentially dissected in abdominal- (aFAT) and perigonadal epididymal- (pEWAT) adipose tissue] and testes from unexposed (CTRL) and BPA-exposed mice sacrificed to 78 *dpp*, were analysed to quantify BPA, T and E2 levels. The BPA concentration (expressed as ng/g) was not significantly different in aFAT and testis in the two experimental groups, while in pEWAT we observed increased BPA concentration in exposed animals compared to the CTRL group (Figure 1A).

The concentrations of T and E2 (expressed as ng/g) were not significantly different in aFAT and pEWAT between CTRL and exposed group (Figure 1B,C). In the testis, T concentration decreased in BPA-exposed mice compared to the CTRL (Figure 1B; *p* < 0.01), while E2 testicular levels were significantly higher (*p* < 0.01) in animals exposed to BPA compared to the CTRL group (Figure 1C).

### 2.2. BPA Effects on Steroidogenesis Enzymes

**Figure 2:**
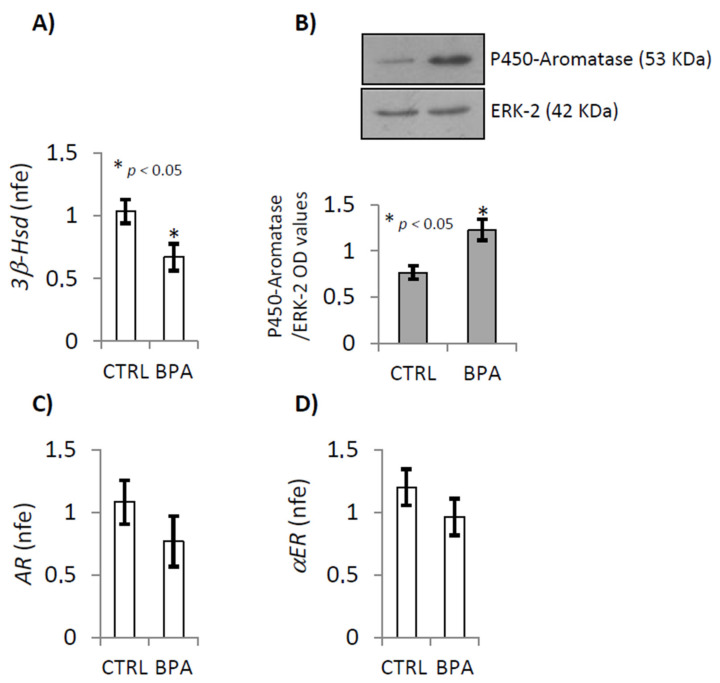
RTqPCR analysis of testicular *3β-Hsd* (**A**), *AR* (**C**) and *αER* (**D**) mRNA in CTRL and BPA exposed mice. Transcripts amounts were reported as normalized fold expression (nfe) relatively to *Rps18* gene (**A**,**C**,**D**). Western Blot analysis of P450-aromatase (**B**) in CTRL and BPA exposed mice. Protein amounts were quantified by densitometry analysis, normalized against ERK-2 signals, and expressed in OD values as fold change (**B**). All data were reported as mean value ± S.E.M. (*: *p* < 0.05).

To better understand the effects of BPA exposure on T and E2, we evaluated the gene expression of relative synthetizing enzymes [3β-hydroxysteroid dehydrogenase (*3β-Hsd*) and P450-aromatase] and receptors [androgen receptor (*AR*), *αER*] using RTqPCR and/or western-blot analyses (Figure 2A–D). BPA exposure caused a significant reduction in testicular mRNA levels of *3β-Hsd* (Figure 2A; *p* < 0.05), while P450-aromatase protein levels were higher in BPA-exposed mice compared to CTRL (Figure 2B; *p* < 0.005). The levels of *AR* and *αER* mRNA were unchanged in testis of mice exposed to BPA in comparison to unexposed controls (Figure 2C,D).

### 2.3. BPA Effects on Endocannabinoid System Components

The expression levels of AEA and 2-AG metabolizing enzymes (NAPE-PLD, FAAH, DAGL and MAGL), as well as of CB1 and CB2 receptors, were investigated in testis from CTRL and BPA-exposed animals by RTqPCR and western blot analyses. Because of the absence of commercially available good antibodies we have not investigated the expression levels of DAGL protein.

**Figure 3:**
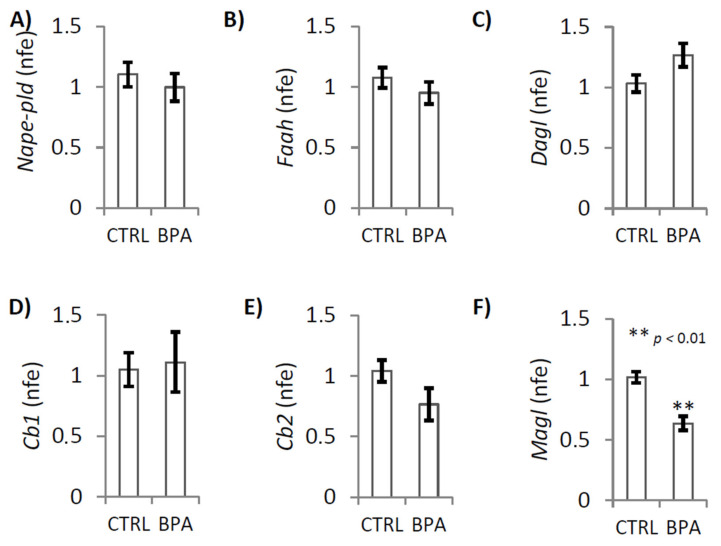
Gene expression analysis of ECS components in testis of male mice early-life exposed to vehicle (CTRL, *n* = 3/4) or BPA (BPA, *n* = 4) and sacrificed at 78 *dpp*. RTqPCR analysis of *Nape-pld*, *Faah*, *Dagl*, *Cb1*, *Cb2*, and *Magl* mRNA. Transcript amounts was reported as normalized fold expression (nfe) relatively to *Rps18* gene (**A**–**F**). All the data were reported as mean value ± S.E.M. (**: *p* < 0.01).

RTqPCR analysis demonstrated that BPA exposure significantly decreased the expression levels of *Magl* mRNA (Figure 3F; *p* < 0.01). No change was observed when we analyzed the expression of *Nape-pld*, *Faah*, *Dagl*, *Cb1* and *Cb2* transcripts (Figure 3A–E).

**Figure 4:**
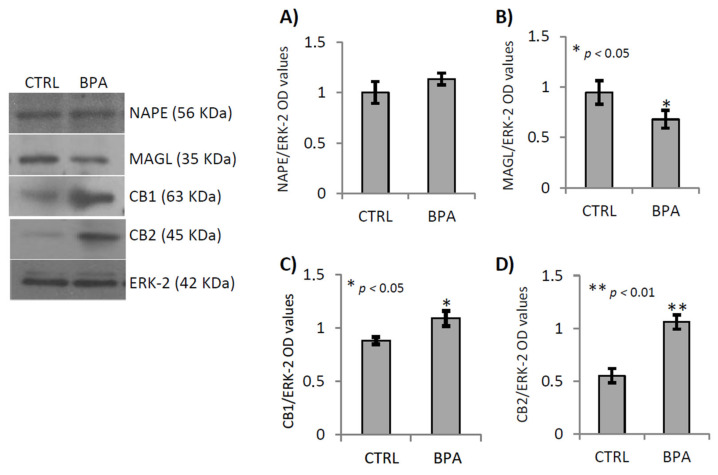
Gene expression analysis of ECS components in testis of male mice early-life exposed to vehicle (CTRL, *n* = 3/4) or BPA (BPA, *n* = 4) and sacrificed at 78 *dpp*. Western Blot analysis of NAPE, MAGL, CB1 and CB2. Protein amounts were quantified by densitometry analysis, normalized against ERK-2 signals, and expressed in OD values as fold change (**A**–**D**). All data were reported as mean value ± S.E.M. (*: *p* < 0.05; **: *p* < 0.01).

Western Blot analysis demonstrated that NAPE-PLD protein (Figure 4A) was equally expressed in both experimental groups while, in BPA exposed animals, there were low levels of MAGL protein (Figure 4B; *p* < 0.05) and highest levels of CB1 and CB2 protein (Figure 4C,D; *p* < 0.05 and *p* < 0.0001 respectively) as compared with CTRL group.

### 2.4. BPA Effects on Germ Cell Progression

CTRL and BPA-exposed testes were used to study the effects of BPA exposure on germ cell progression by western blot analysis. Specific germ cell markers were used to selectively verify the content of mitotic (spermatogonia, SPGs), meiotic (spermatocytes, SPCs) and post-meiotic (spermatids, SPTs) cells. In particular, the VASA protein was used to identify all the germ cells, from SPGs-to-SPTs [76], while the MAGE-A4, SCP-3 and TNP2 proteins were used respectively to identify SPGs and preleptotene/pre-pachytene SPCs, as well as meiotic SPCs and SPTs, respectively [43,77,78].

**Figure 5:**
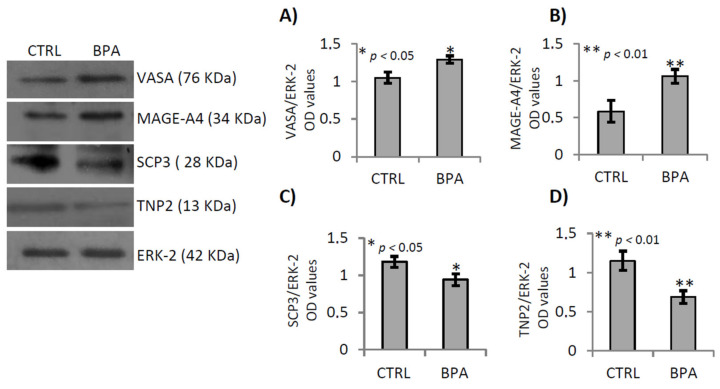
Gene expression analysis of germ cells markers in testis of male mice early-life exposed to vehicle (CTRL, *n* = 3/4) or BPA (BPA, *n* = 4) and sacrificed at 78 *dpp*. Western Blot analysis of VASA, MAGE-A4, SCP3 and TNP2. Protein amounts were quantified by densitometry analysis, normalized against ERK-2 signals, and expressed in OD values as fold change (**A**–**D**). All data were reported as mean value ± S.E.M. (*: *p* < 0.05; **: *p* < 0.01).

Western blot analyses revealed highest content of VASA (Figure 5A; *p* < 0.05) and MAGE-A4 proteins (Figure 5B; *p* < 0.01), in testis of BPA exposed animals while a decrease of SPC3 and TNP2 content was observed (Figure 5C,D; *p* < 0.05 and *p* < 0.01, respectively), suggesting that BPA exposure increased germ cell content, specifically SPGs while it reduced that of SPCs and SPTs. Noteworthy, these proteins are often described as specific markers of male germ cells and to date no effects of BPA on the regulation of their gene expression are known.

**Figure 6:**
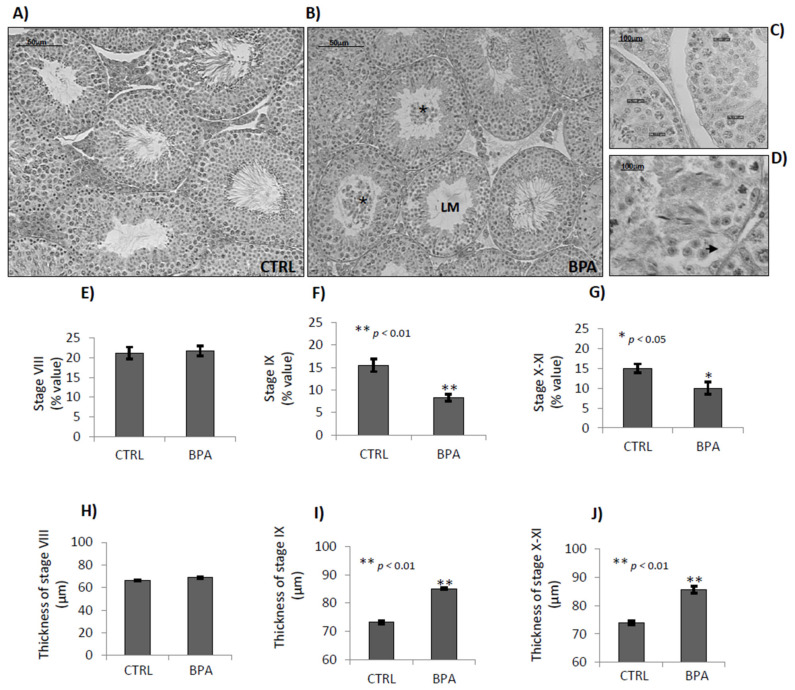
Histological analysis of seminiferous tubules from male mice early-life exposed to vehicle (CTRL) or BPA and sacrificed at 78 *dpp*. Representative H&E stained sections of testis from CTRL (**A**), or BPA (**B**–**D**). LM: Lumen; *: lumen filled with detached germ cells; Arrow: cytoplasma retraction of Sertoli cells (scale bar: 50 μm; inset scale bar: 100 μm). Number of stages VIII (**E**), IX (**F**) and X–XI (**G**) relatively to total tubules (% values). Thickness of seminiferous epitelium (μm) of stage VIII (**H**), IX (**I**) and X–XI (**J**) tubules. All the data were reported as mean values ± S.E.M. (*: *p* < 0.05; **: *p* < 0.01).

The histological analysis of testes revealed that BPA exposure affected the integrity of germinal epithelium and modified the temporal progression of germ cells. The early stages of epithelial cycle frequently showed poorly condensed SPTs in germinal epithelium and desquamated cells within the lumen, notably round and condensed SPTs, suggesting negative effects of BPA on chromatin condensation of SPTs, cell-to-cell junctions (Sertoli/germ cell and/or Sertoli/Sertoli cell) and degeneration of SPTs and SPZs. Some tubules also showed cytoplasm retraction of Sertoli cells (Figure 6A–D).

In BPA exposed animals, the late stages of epithelial cycle (stages VIII-to-XI) appeared rarely. To evaluate temporally and qualitatively their progression, we analysed the percentage and diameter of tubules at stages VIII, IX and X–XI.

No BPA effects were observed on tubules at stage VIII (Figure 6E). The tubular frequency of stages IX (*p* < 0.01) and X–XI (*p* < 0.05) reduced in BPA exposed animals, by almost 40% compared to the CTRL group (Figure 6F,G) and a significative increase of their epithelial diameter was observed (Figure 6I,J; *p* < 0.01) indicative of an accelerated progression of germ cells, specifically SPCs and elongating/condensing SPTs, and epithelial trophia.

### 2.5. BPA Effects on Junctional Proteins

To investigate the adverse effect of BPA on germ cell detachment, we carried out gene expression analyses of some proteins, or mRNA, organizing or modulating cell–cell junctional complexes, particularly proteins related to tight junctions, gap junctions, basal ectoplasmic specialization and emi/desmosome in testis from CTRL and BPA-exposed male at 78 *dpp*, using RT-qPCR or western blot analysis. Specifically, i) the integral membrane proteins such as occludin (*Ocln*), claudin-3 (*Cldn-3*), claudin-5 (*Cldn-5*), connexin-43 (*Cx43*), junctional adhesion molecule 1 (*Jam-1*); ii) the adaptors such as zona occludens protein-1 (*Zo-1*); iii) the actin regulatory proteins such as actin-related protein 2/3 complex subunit 1B (*Arpc1b*).

**Figure 7:**
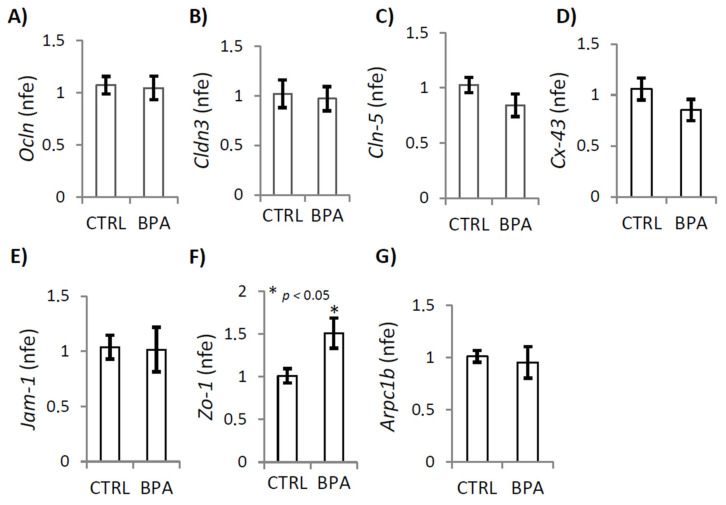
Gene expression analysis of some proteins organizing or modulating cell–cell junctional complexes in testis of male mice early-life exposed to vehicle (CTRL, *n* = 3/4) or BPA (BPA, *n* = 4) and sacrificed at 78 *dpp*. RTqPCR analysis of *Ocln*, *Cldn-3, Cldn-5, Cx43, Jam-1, Zo-1* and *Arpc1b* mRNA. Transcripts amounts were reported as normalized fold expression (nfe) relatively to *Rps18* gene (**A**–**G**). All the data were reported as mean value ± S.E.M. (*: *p* < 0.05).

Results showed that BPA exposure significantly increased *Zo-1* mRNA expression (Figure 7F; *p* < 0.05), while no change was observed when we analyzed the expression of the other genes (Figure 7A–G).

**Figure 8:**
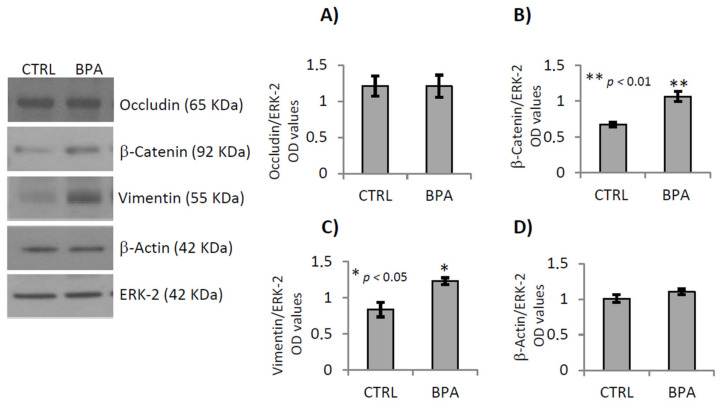
Gene expression analysis of some proteins organizing or modulating cell–cell junctional complexes in testis of male mice early-life exposed to vehicle (CTRL, *n* = 3/4) or BPA (BPA, *n* = 4) and sacrificed at 78 *dpp*. Western Blot analysis of occludin, β-catenin, vimentin and β-actin. Protein amounts were quantified by densitometry analysis, normalized against ERK-2 signals, and expressed in OD values as fold change (**A**–**D**). All data were reported as mean value ± S.E.M. (*: *p* < 0.05; **: *p* < 0.01).

However, β-catenin and vimentin protein levels were higher in BPA-exposed mice than in CTRL group (Figure 8B *p* < 0.01 and Figure 8C *p* < 0.05, respectively). No change was observed when we analyzed the expression of occludin and actin proteins (Figure 8A,D).

## 3. Discussion

Although the adverse effects of BPA on spermatogenesis and SPZs are consistent, there is insufficient evidence to explain how BPA target spermatogenesis and interferes with sperm health/quality parameters. The reported crosslink between steroids and ECS [42] suggests the hypothesis, here verified, that early exposure to BPA induces its accumulation in steroidogenic tissues of adult animals with increase of E2 and adverse effects on spermatogenesis via ECS.

Tissues collected from adult male mice early exposed to BPA, during foetal/perinatal period, were previously used for studies on biology of food intake and epididymal sperm maturation [17,36,42], here used to verify our hypothesis.

In agreement with effective BPA accumulation we analysed T and E2 levels in testis from CTRL and BPA-exposed mice and in two specific fat pads, aFAT and pEWAT, as both are steroidogenic tissues accumulating BPA with a key role of pEWAT in supporting spermatogenesis [40].

In CTRL animals, no BPA was detectable in the testis while scant but measurable amounts of BPA were detected in the fat samples, highlighting the real technical difficulties in avoiding environmental contamination of BPA that as a fat-soluble chemical, preferentially accumulates in adipose tissue. In BPA-exposed animals, the BPA was present in all the tissues analyzed in adulthood. Indeed, although mice become able to metabolize BPA in the pubertal period [16], measurable BPA amounts were detected at 78 *dpp* in all the tissues of exposed animals, with values tending to increase in testis and aFAT, or being significantly highest in pEWAT. Consistently, we noticed that BPA targets adiposity of pEWAT [42]. Therefore, this preferential accumulation phenotypically affects the epididymal fat pad suggesting potential interference with epididymal-fat derived key factors that locally act on ipsilateral testis via pampiniform plexus. Among pEWAT-derived molecules candidate as spermatogenesis modulators there are steroids, including T and estrogens, long chain fatty acid, leptin and grelin [39,79].

Anyway, in exposed animals, there was a slight increase of BPA and E2 in aFAT. The values were not significantly higher compared to CTRL animals but there was a clear trend towards increasing levels. No change in T levels was observed. However, sustained amounts of BPA were specifically detected in pEWAT. The values were significantly higher in exposed animals than in CTRL group revealing that BPA intercepted preferentially pEWAT rather than aFAT, thus demonstrating selective site-specific accumulation. This accumulation did not significantly affect local T or E2 levels. Indeed, although there was a clear trend towards increasing E2 levels in pEWAT of BPA exposed animals, no significant difference was observed between the experimental groups. This clearly indicated that measurable BPA amounts were accumulated in the tissues during the exposure period, from 10 *dpc* to 31 *dpp*, and stably persisted at least up to 78 *dpp*. Thus, exposure to BPA dose below NOEL value, during the foetal-perinatal period, produces sustained and significant BPA accumulation specifically in pEWAT, becoming a risk factor for the endocrine pathways in adulthood. In agreement, significant effects of such BPA accumulation on testicular T and E2 production were observed. In fact, in CTRL testis both BPA and E2 were undetectable while high T levels were observed. However, in exposed animals, BPA become detectable in the testis, with values comparable to those of the CTRL fat tissues. Furthermore, highest E2 levels and lowest T levels were observed, suggesting that the real accumulation of BPA, preferentially in pEWAT, was related to T-E2 conversion in the testis. Gene expression analysis carried out on testis from CTRL and BPA exposed mice, confirmed that exposure to BPA reduced the *3β-Hsd* and increased the P450-aromatase, explaining respectively T and E2 levels. No effect was observed on gene expression of *AR* and *αER* receptors, collectively indicating that BPA exposure increased intratesticular estrogens by targeting T synthesis and aromatization. Consequentially BPA exposure affected the AR-/ER-signalling by modulating the amount of the corresponding ligands, thereby amplifying and reducing estrogenic and androgenic activity, respectively. Interestingly, in BPA exposed animals, the amount of testicular E2 was approximately 100 times greater than the physiological CTRL values, suggesting huge amplification of endogenous estrogen activity in response to BPA exposure. Thus, it was conceivable that BPA accumulation, with its estrogenic activity, could affect ECS impairing spermatogenesis.

Gene expression analysis, confirmed that BPA exposure significantly decreased the expression of the 2-AG hydrolase MAGL, both mRNA and protein, while CB1 and CB2 protein increased. Consistently, testicular CB2 activity locally upregulates expression of CB1 and decreases MAGL expression [66] suggesting that BPA exposure sustained levels of 2-AG, MAGL-dependent, and via hyperexpression of CB2 sustained the activation of 2-AG/CB2 signaling with adverse effects on germ cell activities. In agreement, Zhao and coworkers recently reported that MAGL inhibition, related to 2-AG increase, is mediated by CB1 [80]. However, hyper-activation of CB2 in vivo forces onset of spermatogenesis and accelerates temporal progression of spermatogenesis [65]. With this in mind, we verified the spermatogenesis progression comparing CTRL and BPA-exposed testis at molecular and morphological levels.

In agreement with the observed MAGL and CB2 levels, gene expression analysis of germ-cell specific markers revealed that BPA exposure increased the germ cell content, and especially SPGs, while reducing SPCs and SPTs. This observation was consistent with lowest MAGL and highest 2-AG/CB2 levels observed in murine SPGs as well as with recent finding reported in BPA-exposed zebrafish showing that lowest levels of testicular 2-AG were associated to a lowest number of SPGs [81]. In mouse germ cells there is a decreasing 2-AG/CB2 gradient from SPGs-to-SPTs [56] and in vivo studies highlight the importance of proper CB2 signalling to maintain the correct temporal progression of spermatogenesis [65].

In agreement with germ cell content, the histological observation of the testes showed that BPA modified temporal progression of germ cells and affected integrity of germinal epithelium. In animals exposed to BPA, tubules at late stages of epithelial cycle (stages VIII-to-XI) appeared rarely. To evaluate their progression, both temporally and qualitatively, we analyzed the percentage and diameter of tubules at stages VIII, IX and X–XI. No BPA effects was observed on stage VIII. The frequency of stages IX and X–XI reduced in BPA exposed animals, by almost 40% compared to the CTRL group, and a significant increase of their epithelial diameter was observed, clear indication of an accelerated progression of germ cells, specifically SPCs and elongating/condensing SPTs, and epithelial trophia.

Interestingly, early stages of epithelial cycle, frequently showed poorly condensed SPTs in germinal epithelium and desquamated cells within the lumen, notably round and condensed SPTs, suggesting negative effects of BPA on chromatin condensation of SPTs, cell-to-cell junctions (Sertoli/germ cell and/or Sertoli/Sertoli cell) and degeneration of SPTs and SPZs. However, it is interesting that ECS modulates chromatin condensation of SPTs [43,44,45,67,68] while BPA and dyethildisbestrol, a potent sintetic estrogen, both induce SPG proliferation) [82]. Also, BPA deranges the ECS in Sertoli cells [83] and aromatase-dependent pathway counteracts pro-apoptotic ECS activity on Sertoli cells [83]. Consistently, estrogens are important to maintain the correct Sertoli/germ cell ratio, in the seminiferous epithelium [84]. Thus, the observed production of E2 in animals exposed to BPA, here demonstrated, could interfere with ECS and, in combination with higher SPG content, to affect the Sertoli/germ cell ratio thus promoting exfoliation of more mature SPGs-associated germ cells, i.e., round/condensed SPTs and SPZs.

This observation well explain the observed decrease in SPTs and death effects of BPA on SPZs previously described in caput epididymis [36] and frequently reported in animals exposed to BPA or treated with high doses of E2 [85]. This reinforce our idea that the adverse BPA effects on spermatogeneis are at least in part mediated by its estrogenic activity on ECS.

To investigate the adverse effect of BPA on detachment of germ cells, we carried out gene expression analyses of some proteins organizing or modulating cell–cell junctional complexes, particularly proteins related to tight junctions, gap junctions, basal ectoplasmic specialization and emi/desmosome. Results showed that BPA exposure significantly increased *Zo-1* mRNA expression, while no changes were observed when we analyzed the expression of tight-junction genes. Conversely, levels of the vimentin and β-catenin proteins were higher in BPA-exposed mice than in CTRL group. However, the vimentin-based desmosomes are the anchoring junctions, Sertoli cells-germ cells, from SPGs-to-round SPTs. Subsequently, the actin-based apical ectoplasmic specializations replace the desmosomes and characterizes the anchoring device, between Sertoli cell and elongating SPTs. The junctional complex of apical ectoplasmic specializations include Zo-1 and through the α/β catenin interact with cytoskeletal actin of Sertoli cells [86,87]. The β-catenin conditional deletion in post meiotic cells reveals its key role in survival and chromatin condensation of post-meiotic cells [88] suggesting that BPA reduced post meiotic cells by affecting β-catenin protein. Consistently, it was reported that BPA exposure during foetal-perinatal period, increased β-catenin levels and affected testicular cell development predominantly via Wnt/β-catenin signaling pathway [89].

In conclusion, we show that exposure to BPA dose below NOEL value, during foetal-perinatal period, produces sustained and significant BPA accumulation, specifically in pEWAT, becoming a risk factor for the reproductive endocrine pathways in adulthood. Indeed, BPA promote intratesticular aromatization of T with sustained increase of E2 and T reduction thus revealing the agonistic estrogenic activity of BPA already reported in our animals. We provide evidence that the adverse BPA effects on spermatogeneis are at least in part mediated by ECS.

## 4. Material and Methods

### 4.1. Experimental Design, BPA Exposure, Parameters and Tissue Collection

Here we used tissue collected from the same batch of animals exposed to BPA as already described [17,36]. Briefly, CD1 strain male offspring were daily exposed to drinking water containing BPA (10 µg/mL BPA dissolved in 0.2% ethanol; *n* = 12, exposed group) or vehicle (ethanol 0.2%; *n* = 8, unexposed/control group; CTRL), from 10 *dpc* to 31 *dpp*, and sacrificed in adulthood. Specifically, animals were exposed to BPA from 10 *dpc* to 21 *dpp*, via pregnant/lacting mothers, followed by direct access to water, from 21-to-31 *dpp*. After weaning, each male litter (*n* = 5 litters/CTRL group; *n* = 5 litters/BPA-exposed group) was housed in a single cage and some physiological parameters were constantly monitored. At 78 *dpp* all the animals were sacrificed under anaesthesia and subjected to tissue collection. Testes were processed for molecular and/or histomorphologic analyses. The visceral fat mass were accurately collected and integrally stored or dissected in abdominal fat pad (aFAT) and pEWAT and then stored and processed.

The experimental design was structured to avoid or limit undesired environmental contamination of BPA. Accordingly, standard polypropylene cages (Tecniplast S.p.A., Varese, Italy), corncob bedding (Envigo srl, Udine, Italy) and glass bottles (Zooplus AG, Monaco di Baviera, Germany) were used [22]. The water intake of mothers and offspring was constantly monitored to calculate daily BPA intake and verify that exposure was within NOEL limits. The exposure period was planned to cover full development of germ cells, from gonocytes-to-SPTs [90,91], as well as differentiation and proliferation of Sertoli [90] and adult Leydig cells [70].

Experiments were approved by the Italian Ministry of Education and the Italian Ministry of Health with authorization n 941/2016-PR issued on 10 October 2016. Procedures involving animal care were carried out in accordance with National Research Council’s publication Guide for Care and Use of Laboratory Animals (National Institutes of Health Guide).

### 4.2. Determination of BPA, 17-β-Estradiol and Testosterone Levels in Adipose and Testicular Tissues

Detailed analytical methods for determining BPA, E2 and T levels in adipose (aFAT and pEWAT) and testicular tissues have been published [17], including the quality control system used to monitor method performance and to prevent analysis contamination.

Briefly, samples were subjected to liquid-liquid extraction coupled to a solid phase extraction on AFFINIMIP^®^ SPE ESTROGENS cartridges (Polyntell SA, Paris, France). The analysis of sample extracts was carried out by a Dionex UltiMate 3000 HPLC system (Thermo Fisher Scientific Inc, Rodano, Italy) coupled to a triple quadrupole mass spectrometer (API 2000, AB Sciex, Darmstadt, Germany). A Kinetex F5 (100 × 4.6 mm, 2.6 μm) stainless steel column (Phenomenex, Bologna, Italy) was used for reversed-phase separations. The chromatographic separation and instrumental parameters were reported in Errico et al. [17]. The analytes were quantified in multiple reaction monitoring mode. All samples were analysed in triplicate with relative standard deviations (RSDs) less than 13%.

### 4.3. Total RNA Preparation

Total RNA was extracted from testis (*n* = 4 for CTRL group; *n* = 4 for BPA exposed group) using TRIZOL Reagent (Invitrogen Life Technologies, Monza, Italy) in agreement with manufacturer’s instructions. RNA samples (10 µg) were treated with 1 µL Deoxyribonuclease (DNAse, 10 U/µL) at 37 °C for 10 min and further processed accordingly to manufacturer’s instructions (GE Healthcare, Milano, Italy). Total RNA purity and integrity were determined by spectrophotometry at 260/280 nm and by electrophoresis.

### 4.4. cDNA Synthesis and Quantitative Real Time-PCR (qRT-PCR)

In agreement with manufacturer’s instructions (Life Technologies, Monza, Italy), cDNA synthesis was carried out in 20 μL final volume containing 1× first strand buffer, 5 μg of total RNA-DNA free, 0.5 μg oligo dT_(18)_, 0.5 mM dNTP mix, 5 mM DTT, 40 U RNase Out (Life Technologies), 200U SuperScript-III RnaseH^-^ Reverse Transcriptase (Life Technologies). As negative control, cDNA was synthesized as above detailed without to add the reverse transcriptase enzyme.

qRT-PCR analysis was performed according to the manufacturer’s instructions (CFX-96; Bio-Rad, Milano, Italy) in a 20 µL reaction mixture (Syber Green; Bio-Rad) containing diluted cDNA (1:5). Assays were performed in triplicate, and a standard curve from consecutive 5-fold dilutions (2 µg–31 ng) of a cDNA pool representative of all samples was included for PCR efficiency determination. Relative gene expression analysis, corrected for PCR efficiency and normalized toward reference gene (ribosomal protein S18, *Rps18*) was performed by CFX Manager software (Bio-Rad). For details about genes, primer sequences, annealing temperatures and product size see Table 1. Results were expressed as mean value of normalized fold expression (nfe) ± S.E.M.

### 4.5. Protein Extraction and Western Blot Analysis

Testes from CTRL and BPA exposed mice were sonicated in RIPA buffer [PBS, pH 7.4, 10 mM dithiothreitol, 0.02% sodium azide, 0.1% SDS, 1% Nonidet P-40, 0.5% sodium deoxycholate, in the presence of protease inhibitors (10 μg/mL of leupeptin, aprotinin, pepstatin A, chymostatin, and 5 μg/mL of TPCK)] and analyzed by western blot. Briefly: proteins were separated by SDS-PAGE (10% acrylamide) and transferred to polyvinylidene difluoride membrane (GE Healthcare, Milano, Italy) at 280 mA for 2.5 h at 4 C. Filters were treated for 3 h with blocking solution [5% nonfat milk, 0.25% Tween-20 in Tris-buffered saline (TBS, pH 7.6)] and then incubated overnight, at 4 °C in TBS-milk buffer (TBS pH 7.6, 3% nonfat milk) with different primary antibody After washing in 0.25% Tween20-TBS, filters were incubated with the secondary antibodies, diluted 1:1000 in TBS-milk buffer and then washed again. The immune complexes were detected using the enhanced chemiluminescence-Western blotting detection system (Amersham ECL Western Blotting Detection Reagent; GE Healthcare, Milano, Italy). Antibodies and relative dilutions are reported in Table 2.

Signals were quantified by densitometry analysis, adjusted relatively to ERK1/2 levels and expressed in optical density (OD) fold change (mean ± S.E.M.). The specificity of immunoreaction has already been demonstrated [44,74,92,93] and here routinely checked by omitting primary antibody.

### 4.6. Testicular Morphology, Spermatogenetic Stage and Tubular Thickness Analysis

Testis from CTRL (*n* = 4) and BPA-exposed (*n* = 4) animals was fixed in Bouin’s solution for 12 h, dehydrated in ethanol, cleared in xylene and embedded in paraffin. Serial sections (10 μm) were cut and processed for histological analysis. For hematoxylin-eosin (H&E) stain, the section were deparaffinized and rehydrated (2 × 5′ xylene; 2 × 5′ 100% ethanol; 1 × 5′ 95% ethanol; 1 × 5′ 85% ethanol; 1 × 5′ 75% ethanol and 1 × 5′ deionized H_2_O). Successively, the sections were processed for Hematoxalin (1 × 5′) and Eosin staining (1 × 3′) and dehydratated (1 × 3′ 75% ethanol; 1 × 3′ 85% ethanol; 1 × 3′ 95% ethanol; 2 × 5′ 100% ethanol; 2 × 5′ xylene).

Images were captured using a high-resolution digital camera (DC300F; Leica Microsystems Inc., Milan, Italy) and, in combination with the direct observation at microscope, these were used to study: (i) the spermatogenetic stages relative to early meiotic phase of prophase I (i.e., stages VIII up to XI); (ii) the thickness of the seminiferous epithelium (µm) relatively to spermatogenetic stages VIII, IX and X–XI. Serial sections were specifically used to properly identify the spermatogenetic stages. The identification of stages VIII-to-XI was based on specific features of SPCs and SPTs as previously reported [43,91]. In particular, tubules at stage VIII were identified by the presence of SPZs lining the luminal surface of the seminiferous epithelium with tails addressed to the lumen. The identification of tubules at stages IX and grouped stages X–XI were based on big size of pachytene SPCs and specifically discriminated by nuclear shape of SPTs and relative chromatin condensation state (step 9, 10 and 11 of spermiogenesis, respectively). Testicular sections from CTRL and BPA exposed mice were used to identify and count tubules at stages VIII (containing _PL_SPCs), IX (containing _L_SPCs) and X–XI (containing _L/Z_SPCs), as well as to count total tubules. A total of 610 tubules from four different CTRL testes and 750 tubules from four different BPA testes were examined and used to calculate the percentage of tubules at stages VIII, IX and X–XI.

Testicular sections from CTRL and BPA exposed mice were also used to evaluate thickness of the seminiferous epithelium (µm) of tubules at spermatogenetic stages VIII, IX and X–XI through the IM100 software using a measurement module tool (Leica Microsystems Inc.).

As routinely required for this experimental procedures, all the results were validated two times by the same operator. The percentage of tubules at stage VIII, IX and X–XI and the thickness of the seminiferous epithelium (µm) at same stages were reported as mean values ± S.E.M.

### 4.7. Statistical Analysis and Data Presentation

Student’s *t*-test and Duncan’s test (for multi group comparison) were carried out to evaluate the significance of differences. Data are expressed as the mean ± S.E.M. from at least 3–6 independent animals/samples for each experimental group.

## Figures and Tables

**Table 1:** Primer sequences (S: sense; AS: antisense), annealing temperature (Tm) and product size (bp) used for testicular gene expression analyses in CTRL and BPA exposed mice by Real-Time qPCR.

Gene Primers	Sequenses 5′–3′	Tm (°C)	Product Size (bp)
*AR S*	gacctgcctgatctgtgg	58	206
*AR AS*	gagtcatccctgcttcataa		
*aER S*	tgccctactacctggagaa	58	170
*aER AS*	gtagcgagtctccttggc		
*3b-Hsd S*	agtattccgaccagaaaccaag	60	75
*3b-Hsd AS*	atccagaatgtctccttccaac		
*Ocln S*	cctactcctccaatggcaaa	55	208
*Ocln AS*	ctcttgccctttcctgcttt		
*Cldn-3 S*	gcacccaccaagatcctcta	57	205
*Cldn-3 AS*	tcgtctgtcaccatctggaa		
*Cldn-5 S*	agagcagaggcaccagaatc	57	143
*Cldn-5 AS*	acacagcaccagacccagaa		
*Cx-43 S*	ctttgacttcagcctccaag	54	176
*Cx-43 AS*	gaaaatgaagagcaccgaca		
*Jam-1 S*	cactgattctccttggactctt	56	157
*Jam-1 AS*	gaacgacgaggtctgtttgaa		
*Zo-1 S*	gcaccatgcctaaagctgtc	57	122
*Zo-1 AS*	actcaacacaccaccattgc		
*Arpc1b S*	agctgatgtttgaatcgagc	56	188
*Arpc1b AS*	tttctgtgatgaaggtgacg		
*Nape-pld S*	tggtttatgaaataccagca	56	159
*Nape-pld AS*	atctcttcaaaagcggg		
*Faah S*	agattgagatgtatcgccag	56	260
*Faah AS*	cttcagaatgttgtcccac		
*Dagl S*	atcactgtcctctgcgtctt	54	202
*Dagl AS*	tttctgagtaggcatctgact		
*Magl S*	ggccctcatctttgtgtcc	60	168
*Magl AS*	ctgacgaaaacgtggaagtc		
*Cb1 S*	ctgatcctggtggtgttgat	60	162
*Cb1 AS*	cctcagagcatagatgatgg		
*Cb2 S*	aacggtggcttggagttcaa	57	177
*Cb2 AS*	gaacaggtacgagggctttct		
*Rps18 S*	gagactctggatgctaactag	56	172
*Rps18 AS*	ggacatctaagggcatcacag		

**Table 2:** Primary antibodies, protein amounts, antibody dilution and secondary antibodies used for western blot analysis.

Primary Antibody	µg of Protein	Antibody Dilution	Secondary Antibody
AROMATASE (Elabscience 31086)	50	1:250	HRP-conjugated rabbit IgG (Dako Corp., Milan, Italy)
OCCLUDIN (Thermo Fisher 40-4700)	70	1:500	HRP-conjugated rabbit IgG (Dako Corp., Milan, Italy)
b-CATENIN (Santa Cruz sc-7199)	70	1:1000	HRP-conjugated rabbit IgG (Dako Corp., Milan, Italy)
VIMENTIN (Elabscience 27405)	70	1:2000	HRP-conjugated mouse IgG (Dako Corp., Milan, Italy)
b-ACTIN (Elabscience 20031)	70	1:2000	HRP-conjugated mouse IgG (Dako Corp., Milan, Italy)
VASA (Abcam ab13840)	20	1:1000	HRP-conjugated rabbit IgG (Dako Corp., Milan, Italy)
MAGE-A4 (Abcam ab139297)	70	1:1000	HRP-conjugated mouse IgG (Dako Corp., Milan, Italy)
SCP3 (Invitrogen PA1-16766)	30	1:1000	HRP-conjugated rabbit IgG (Dako Corp., Milan, Italy)
TNP2 (Santa Cruz sc-21106)	70	1:500	HRP-conjugated goat IgG (Dako Corp., Milan, Italy)
CB1 (produced by Prof. Ken Mackie)	20	1:1000	HRP-conjugated rabbit IgG (Dako Corp., Milan, Italy)
CB2 (Abcam ab45942)	30	1:1000	HRP-conjugated rabbit IgG (Dako Corp., Milan, Italy)
NAPE-PLD (Cayman 101600)	50	1:500	HRP-conjugated rabbit IgG (Dako Corp., Milan, Italy)
MAGL (Abcam ab24701)	80	1:500	HRP-conjugated rabbit IgG (Dako Corp., Milan, Italy)
ERK-2 (Santa Cruz sc-1647)	/	1:1000	HRP-conjugated mouse IgG (Dako Corp., Milan, Italy)

## Data Availability

The datasets generated during and/or analysed during the current study are available from the corresponding author on reasonable request.
